# A Novel Anti-Inflammatory d-Peptide Inhibits Disease Phenotype Progression in an ALS Mouse Model

**DOI:** 10.3390/molecules26061590

**Published:** 2021-03-13

**Authors:** Julia Post, Vanessa Kogel, Anja Schaffrath, Philipp Lohmann, N. Jon Shah, Karl-Josef Langen, Dieter Willbold, Antje Willuweit, Janine Kutzsche

**Affiliations:** 1 Institute of Biological Information Processing, Structural Biochemistry, IBI-7, Forschungszentrum Jülich GmbH, 52425 Jülich, Germany; j.post@fz-juelich.de (J.P.); vkogel@ukaachen.de (V.K.); a.schaffrath@fz-juelich.de (A.S.); 2 Institute of Neuroscience and Medicine 4, INM-4, Medical Imaging Physics, Forschungszentrum Jülich GmbH, 52425 Jülich, Germany; p.lohmann@fz-juelich.de (P.L.); n.j.shah@fz-juelich.de (N.J.S.); k.j.langen@fz-juelich.de (K.-J.L.); 3 Institute of Neuroscience and Medicine 11, INM-11, JARA, Forschungszentrum Jülich GmbH, 52425 Jülich, Germany; 4 JARA-Brain-Translational Medicine, 52074 Aachen, Germany; 5 Department of Neurology, RWTH Aachen University, 52062 Aachen, Germany; 6 Department of Nuclear Medicine, RWTH Aachen University, 52062 Aachen, Germany; 7 Institut für Physikalische Biologie, Heinrich-Heine-Universität Düsseldorf, 40225 Düsseldorf, Germany

**Keywords:** amyotrophic lateral sclerosis, behaviour, d-enantiomeric peptide, neuroinflammation, SOD1*G93A mice

## Abstract

Amyotrophic lateral sclerosis (ALS) is a progressive neurodegenerative disease characterised by selective neuronal death in the brain stem and spinal cord. The cause is unknown, but an increasing amount of evidence has firmly certified that neuroinflammation plays a key role in ALS pathogenesis. Neuroinflammation is a pathological hallmark of several neurodegenerative disorders and has been implicated as driver of disease progression. Here, we describe a treatment study demonstrating the therapeutic potential of a tandem version of the well-known all-d-peptide RD2 (RD2RD2) in a transgenic mouse model of ALS (SOD1*G93A). Mice were treated intraperitoneally for four weeks with RD2RD2 vs. placebo. SOD1*G93A mice were tested longitudinally during treatment in various behavioural and motor coordination tests. Brain and spinal cord samples were investigated immunohistochemically for gliosis and neurodegeneration. RD2RD2 treatment in SOD1*G93A mice resulted not only in a reduction of activated astrocytes and microglia in both the brain stem and lumbar spinal cord, but also in a rescue of neurons in the motor cortex. RD2RD2 treatment was able to slow progression of the disease phenotype, especially the motor deficits, to an extent that during the four weeks treatment duration, no significant progression was observed in any of the motor experiments. Based on the presented results, we conclude that RD2RD2 is a potential therapeutic candidate against ALS.

## 1. Introduction

Alzheimer’s disease (AD), Parkinson’s diseases (PD) and amyotrophic lateral sclerosis (ALS) are among the most common neurodegenerative diseases in adults. In addition to cognitive impairment, selective neuronal death and neuroinflammation in the central nervous system are prominent pathologic features in AD [1,2,3,4]. Clinically, ALS manifests as focal muscular weakness, with atrophy of skeletal muscles up to progressive paralysis and premature death, usually from respiratory failure [5]. Most ALS cases are sporadic (sALS), while a minority are familial cases (fALS) and caused by inherited mutations [6]. A key discovery was the identification of a mutation in the gene of the enzyme superoxide dismutase 1 (SOD1), which is causative in up to 20% of all fALS and in up to 3% of sALS cases [7,8]. To investigate the pathophysiology of ALS and the role of mutated SOD1 in disease development and progression of ALS, a transgenic mouse model was created (tg(SOD1*G93A)1Gur), which expresses mutant SOD1 (SOD1*G93A) and develops adult-onset neurodegeneration of neurons in the lumbar spinal cord and motor cortex and progressive motor deficits, which leads to paralysis [9,10,11,12]. Due to similar clinical features and pathology to human ALS, these mice have been studied extensively over years and are still a cornerstone of preclinical ALS research [13]. In addition to the clinical symptoms, neuroinflammation and immune-inflammatory processes are further prominent pathological hallmarks of human ALS cases and the transgenic SOD1*G93A mice [14,15,16]. Neuroinflammation is characterised by the presence of activated glial cells, mainly microglia and astrocytes. In addition, previous studies demonstrated that activated astrocytes and microglia play a role in disease progression of ALS [17,18]. Cytokines, the primary messengers of inflammatory processes, are released by microglia and astrocytes in response to neuroinflammation [19,20,21]. They can be classified into two different types: type 1 cytokines (= pro-inflammatory) increase the inflammatory reaction, while type 2 cytokines (= anti-inflammatory) decrease the inflammatory reaction. In a non-pathological state, a complex signalling cascade produces a protective immune response through cytokines [22,23]. A temporal increased level of inflammatory cytokines was observed in ALS, but also in AD [24,25,26]. Like many other neurodegenerative diseases, such as AD, to date there is no curative therapy for ALS. Thus far, only symptomatic and minor life-prolonging treatments are available [27,28,29].

Recently, we described the development of compounds for a disease-modifying treatment of AD. The compound RD2 preferentially binds amyloid beta (Aβ) monomers with nanomolar affinity and stabilises Aβ in its native conformation [30], which is a novel strategy to directly disassemble toxic Aβ oligomers into native monomers [31]. RD2 has recently successfully passed a phase I clinical trial in healthy subjects (Single Ascending Doses (SAD) EUDRA-CT: 2017-000396-93 and Multiple Ascending Doses (MAD) EUDRA-CT: 2018-002500-14) [32] after demonstrating its preclinical efficacy in several AD mouse models [33,34,35,36]. The head-to-tail tandem version of RD2, RD2RD2, was originally designed to obtain a bivalent version of RD2 with potentially higher avidity and affinity for polyvalent Aβ assemblies. Like RD2, also RD2RD2 belongs to a relatively new class of drugs, the all-d-peptides, which consist solely of d-enantiomeric amino acid residues and which exhibit several advantages including high proteolytic stability and low immunogenicity [37,38].

Here, we explored the therapeutic potential of RD2RD2 in the ALS SOD1*G93A transgenic mouse model. For this purpose, we treated SOD1*G93A transgenic mice intraperitoneally for four weeks with RD2RD2 vs. placebo and performed longitudinally various behavioural and motor coordination tests. Subsequently, the brain stem and lumbar spinal cord of treated mice were immunohistochemically investigated.

## 2. Results

Earlier, we tested the efficacy of RD2RD2 in an AD specific mouse model overexpressing a double mutation of amyloid precursor protein and presenilin 1 (APP/PS1) by intraperitoneal treatment for four weeks with either RD2RD2 or placebo. Treatment with RD2RD2 had a strong effect on neuroinflammation as it significantly reduced the number of activated microglia in both cortex and hippocampus down to levels of non-transgenic mice (ntg) (Figure S1a, e–g and b, h–j and Table S1). In addition, RD2RD2-treated mice displayed significantly reduced astrogliosis (antibody glial fibrillary acidic protein (GFAP)) in the cortex and hippocampus (Figure S1c, k–m and d, n–p and Table S1). Additionally, analysis of the inflammatory marker levels revealed a remarkable and significant decrease in the levels of all cytokines measured in RD2RD2-treated APP/PS1 mice in comparison to the placebo group (Figure S2a–g and Table S2).

Encouraged by the strong effect of RD2RD2 on neuroinflammation in treated APP/PS1 mice, we performed a treatment study using the transgenic ALS mouse model SOD1*G93A, a model in which neuroinflammation has been described to drive disease progression [18,19].

### 2.1. RD2RD2 Treatment Prevented Further Motor Phenotype Progression in SOD1*G93A Mice

**Figure 1:**
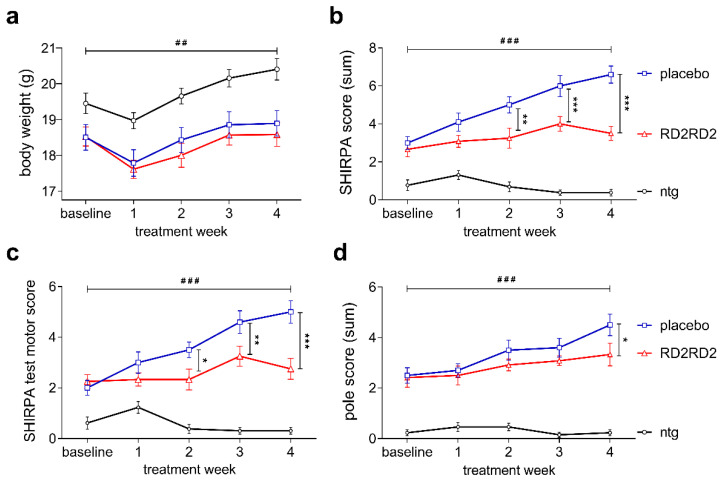
RD2RD2 treatment prevented further significant progression of motor phenotype in SOD1*G93A mice. Changes in absolute body weight (g) over time during treatment revealed significant differences between transgenic and non-transgenic mice (**a**). Analysis of the SHIRPA test battery to evaluate the phenotypic development of RD2RD2- and placebo-treated SOD1*G93A mice resulted in halt of phenotype progression upon treatment (**b**). Subdivision of the SHIRPA parameters into a motor score revealed significant inhibition of motor symptom progression in RD2RD2- vs. placebo-treated SOD1*G93A (**c**). Pole test analysis (**d**) resulted in significant conservation of motor skills in RD2RD2-treated mice, whereas the motor deficits progressed further in placebo-treated mice. All non-transgenic mice exhibited normal motor function throughout the experimental period. Data is presented as mean ± SEM. Statistical calculations were conducted by two-way RM ANOVA with Fisher’s LSD post hoc analysis, non-transgenic mice (ntg) *n* = 13, RD2RD2 *n* = 12 and placebo *n* = 10 for each test. Lozenges and asterisks (*) indicate a significance between treatment groups (ntg vs. RD2RD2 or ntg vs. placebo: ^##^ *p* < 0.01, ^###^ *p* < 0.001 and RD2RD2 vs. placebo: * *p* = 0.05, ** *p* < 0.01, *** *p* < 0.001).

Twelve weeks old female SOD1*G93A mice were treated with placebo (*n* = 10) or 19 mg/kg/d RD2RD2 (*n* = 12) and the non-transgenic littermates were treated with placebo (*n* = 14) formulated in an intraperitoneal (i.p.) osmotic minipump for 28 days. After a small weight loss post-operatively, all mice gained weight continuously over the four weeks treatment period. Throughout the whole testing period the average body weight was significantly different between non-transgenic and transgenic mice (Figure 1a; RD2RD2: 18.8 ± 0.2 g, placebo: 19.0 ± 0.2 g vs. ntg: 20.3 ± 0.3 g; two-way repeated measurement (RM) analysis of variance (ANOVA), F(2,128) = 7.87, *p* = 0.002, Fisher’s least significant difference (LSD) post hoc analysis, ntg vs. RD2RD2 *p* < 0.001 and ntg vs. placebo *p* = 0.006). RD2RD2 treatment neither influenced the average body weight nor body weight gain.

The SmithKline Beecham, Harwell, Imperial College, Royal London Hospital, phenotype assessment (SHIRPA test battery) was used to monitor the progression of the neurodegenerative phenotype of transgenic placebo- or RD2RD2-treated mice [39,40]. At baseline and during treatment, both transgenic groups showed behavioural and motor impairments compared to their non-transgenic littermates (Figure 1b; two-way RM ANOVA, F(2,128) = 101.06, *p* < 0.001, Fisher’s LSD post hoc analysis, ntg vs. RD2RD2 *p* < 0.001 and ntg vs. placebo *p* < 0.001). Already after two weeks of treatment, the difference in SHIRPA score was statistically significant between the RD2RD2 group and placebo-treated SOD1*G93A mice (Figure 1b; RD2RD2 vs. placebo *p* < 0.001).

The subdivision of the SHIRPA parameters into a motor score revealed further specific details about progression of motor deficits of placebo- but not RD2RD2-treated mice (Figure 1c). At the end of the treatment period, motor deficits were significantly lower upon RD2RD2 treatment in comparison to placebo-treated SOD1*G93A mice (Figure 1c; motor score, two-way RM ANOVA, F(2,128) = 66.77, *p* < 0.001, Fisher’s LSD post hoc analysis, placebo vs. ntg *p* < 0.001 and placebo vs. RD2RD2 *p* < 0.001).

Additional investigations on the motor performance of RD2RD2-treated SOD1*G93A mice were performed with the pole test (Figure 1d). This test indicates functional deficits in motor coordination and muscular strength of SOD1*G93A mice. Throughout the experiment, non-transgenic mice did not show any motor deficits, while transgenic SOD1*G93A mice displayed already a significantly higher pole score at baseline (Figure 1d; two-way RM ANOVA, F(2,128) = 104.95, *p* < 0.001, Fisher’s LSD post hoc analysis, ntg vs. RD2RD2 *p* < 0.001 and ntg vs. placebo *p* < 0.001). There was a slight but significant progression in the motor deficit of the placebo group, while the RD2RD2-treated SOD1*G93A mice kept their motor skills at a constant level (Figure 1d; two-way RM ANOVA, F(1,80) = 0.85, *p* = 0.05, Fisher’s LSD post hoc analysis, RD2RD2 vs. placebo (treatment week 4) *p* = 0.021).

**Figure 2:**
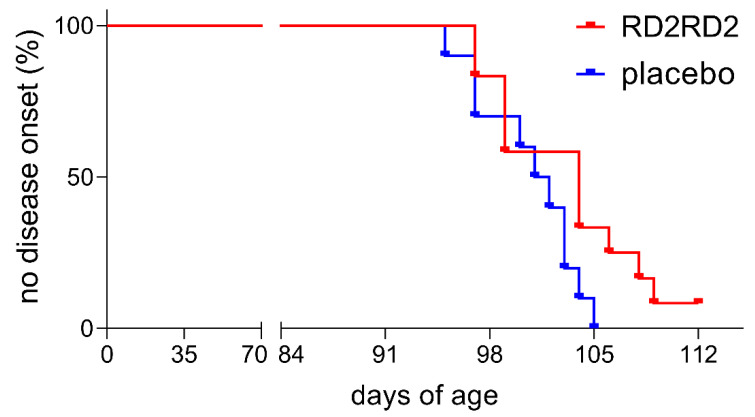
RD2RD2 administration resulted in a non-significant shift of disease onset of SOD1*G93A mice. Assessment of disease was performed three times a week from the start of the experiment. Disease onset is defined as first defects detectable in hind limb splay and an increasing tremor. RD2RD2-treated SOD1*G93A mice had a delayed onset of disease symptoms vs. placebo group. One treated mouse did not develop a tremor in hind limbs until the end of the experiment. Statistical calculations were conducted by Kaplan–Meier survival analysis and the log-rank analysis, RD2RD2 *n* = 12 and placebo *n* = 10.

Assessment of disease onset was measured by the score of Mead et al., 2011 [41]. Treatment delayed symptom onset in RD2RD2- vs. placebo-treated SOD1*G93A mice, however Kaplan–Meier analysis did not reveal a significant difference in disease onset (Figure 2; average disease onset: RD2RD2 104 days and placebo 101.5 days). Within the non-transgenic mice, there was no detectable absence of hind limb splay or tremor during the study.

### 2.2. RD2RD2 Treatment Led to Reduction of Activated Glia Cells and Restored Neuron Density in SOD1*G93A Mice

**Figure 3:**
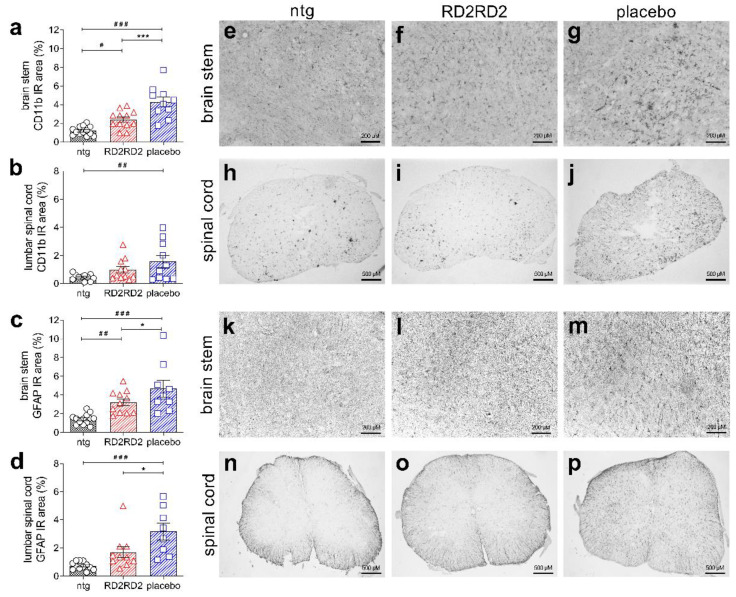
Immunohistochemical investigations revealed a reduction of neuroinflammation in RD2RD2-treated SOD1*G93A mice. Analysis of activated microglia and astrocytes in brain stem and lumbar spinal cord showed significant reduction of neuroinflammation (staining with CD11b and GFAP antibodies with subsequent quantification of glia cells). Presentation of the analysed cells, brain and lumbar spinal cord are given on the right (microglia: **a**,**e**–**g** = brain stem and **b**,**h**–**j** = lumbar spinal cord; astrocytes: **c**,**k**–**m** = brain stem and **d**,**n**–**p** = lumbar spinal cord). Data is presented as mean ± SEM. Statistical calculations were conducted by one-way ANOVA with Fisher’s LSD post hoc analysis, CD11b: ntg *n* = 13, RD2RD2 *n* = 12, placebo *n* = 10 (brain stem) and ntg *n* = 11, RD2RD2 *n* = 11, placebo *n* = 10 (lumbar spinal cord) and GFAP: ntg *n* = 13, RD2RD2 *n* = 11, placebo *n* = 9 (brain stem) and ntg *n* = 12, RD2RD2 *n* = 10, placebo *n* = 8 (lumbar spinal cord). Lozenges (^#^) and asterisks (*) indicate a significance between treatment groups (ntg vs. RD2RD2 or ntg vs. placebo: ^#^ *p* = 0.05, ^##^ *p* = 0.01, ^###^ *p* < 0.001 and RD2RD2 vs. placebo: * *p* = 0.05, *** *p* < 0.001). IR: immunoreactivity. Circles: placebo-treated ntg; triangles: RD2RD2-treated SOD1*G93A mice and squares: placebo-treated SOD1*G93A mice.

To support the findings of the SHIRPA and pole test of RD2RD2-treated SOD1*G93A mice, we analysed activation of inflammatory cells in the brain stem, as well as neuronal nuclei in the motor cortex of all mice. Moreover, we analysed sections of the lumbar spinal cord, since motor weakness is first detectable in the hind limbs in this mouse model and the motoric pathways are linked through the lumbar spinal cord to the brain. For pathological analysis, levels of activated glia cells (using an antibody against integrin α-M/β-2 (CD11b) labelling activated microglia in general, and an antibody against GFAP labelling activated astrocytes) and the number of neurons (using an antibody against neuronal nuclear proteins (NeuN)) were determined by immunohistochemical staining and quantified. Immunolabelling revealed a significantly decreased number of activated microglia in the brain stem of RD2RD2- vs. placebo-treated SOD1*G93A mice (Figure 3a,e–g and Table 1). In lumbar spinal cord sections from RD2RD2-treated SOD1*G93A mice, activated microglia also showed a decrease which did not reach statistical significance towards placebo-treated animals (Figure 3b,h–j and Table 1). Additionally, we analysed activated astrocytes. There was a significant difference in the number of activated astrocytes in the brain stem between all three treatment groups after 28 days of treatment (Figure 3c,k–m and Table 1). RD2RD2 treatment was able to reduce also the number of activated astrocytes significantly in comparison to the placebo group. More important, analysis of activated astrocytes within the lumbar spinal cord revealed a significant decrease in the RD2RD2-treated SOD1*G93A mice down to levels which are not significant to non-transgenic littermates (Figure 3d,n–p and Table 1).

**Figure 4:**
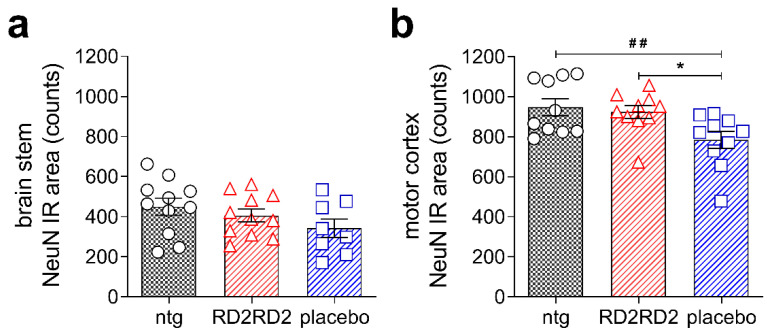
Treatment with RD2RD2 rescued significantly neurons in the motor cortex of SOD1*G93A mice. Analysis of neurons in the brain stem (**a**) and motor cortex (**b**) revealed a significant loss in placebo-treated SOD1*G93A mice, while the count of neurons in RD2RD2-treated mice was similar to the count of non-transgenic mice. Data is presented as mean ± SEM. Statistical calculations were conducted by one-way ANOVA with Fisher’s LSD post hoc analysis, ntg *n* = 11, RD2RD2 *n* = 11, placebo *n* = 8 (brain stem) and ntg *n* = 10, RD2RD2 *n* = 10, placebo *n* = 10 (motor cortex). Lozenges and asterisks (*) indicate a significance between treatment groups (ntg vs. RD2RD2 or ntg vs. placebo: **^##^** *p* = 0.01 and RD2RD2 vs. placebo: * *p* = 0.05). IR: immunoreactivity. Circles: placebo-treated ntg; triangles: RD2RD2-treated SOD1*G93A mice and squares: placebo-treated SOD1*G93A mice.

Moreover, we analysed the number of neurons in SOD1*G93A mice in the brain stem and motor cortex (Figure 4a,b, and Table 1). Quantification of NeuN-positive cells revealed a loss of neurons in transgenic SOD1*G93A mice vs. non-transgenic littermates in both regions. Treatment with RD2RD2 significantly rescued neurons in the motor cortex to levels of non-transgenic littermates. The number of neurons in the brain stem showed the same, but non-significant, tendency (Table 1).

Immunohistochemical staining of neurons was also performed for the lumbar spinal cord, but due to a sub-optimal staining protocol, the neuronal count could not be quantified.

**Figure 5:**
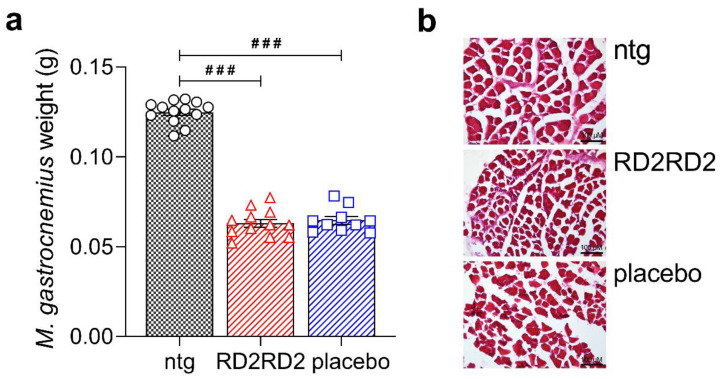
Analysis of the *M. gastrocnemius* in SOD1*G93A mice. Weighing of the *M. gastrocnemius* of both hind limbs revealed a significant difference between non-transgenic and transgenic mice, but not between treatment groups (**a**). A first staining of muscle slices was conducted with haematoxylin and eosin (H&E) to assess muscle degeneration (**b**). Data is presented as mean ± SEM. Statistical calculations were conducted by one-way ANOVA with Fisher’s LSD post hoc analysis, ntg *n* = 13, RD2RD2 *n* = 12 and placebo *n* = 10. Lozenges indicate a significance between treatment groups (ntg vs. RD2RD2 or ntg vs. placebo: ^###^ *p* < 0.001). Circles: placebo-treated ntg; triangles: RD2RD2-treated SOD1*G93A mice and squares: placebo-treated SOD1*G93A mice.

At the end of the study, muscles were harvested along with brain and spinal cord tissues. We investigated the *M. gastrocnemius* of the hind limbs of all mice to determine a potential treatment effect on muscle degeneration (Figure 5). Analysis of the muscle revealed a significant difference between non-transgenic and transgenic mice, but not between treatment groups (Figure 5a). Further, muscle degeneration of the *M. gastrocnemius* was visualised by haematoxylin and eosin (H&E) staining. Myofibrils of the non-transgenic mice were rectangular and regularly shaped, while first irregularly-shaped structures of myofibrils of both transgenic groups were detectable (Figure 5b).

## 3. Discussion

ALS and AD are both fatal neurodegenerative diseases affecting the central nervous system. Despite intensive research, current treatment options are only symptomatic [29,42]. Neuroinflammation plays a major role in both diseases, increasing evidence has firmly certified that neuroinflammation induced by SOD1 plays a key role in ALS pathogenesis [14,25], as well as induced by Aβ in the pathogenic process in AD [43,44].

Based on promising anti-inflammatory effects in an earlier study with AD mice (fully reported in the Supplementary Materials), we examined the compound RD2RD2 for its efficacy in the treatment in a mouse model of a neurodegenerative disorder in which neuroinflammation is known as the main driver of disease. Therefore, the SOD1*G93A ALS mouse model on a C57Bl/6 genetic background was chosen [17,18,26,45]. These mice exhibit the first measurable deficits in motoric performance at age 8.5 weeks [41], which is by definition within the pre-symptomatic phase [46]. Using the SHIRPA test battery, we were able to confirm these early motor deficits of the mouse line in house (Figure S3). Disease onset, defined as the first signs of tremor and hind limb splay defects, started in our colony at an average of week 14 (Figure 2). At the age of 12 weeks, intraperitoneal treatment with RD2RD2 or placebo was initiated in SOD1*G93A mice, which is approximately two weeks before disease onset. RD2RD2 treatment of SOD1*G93A mice significantly slowed their phenotype progression, as measured using the SHIRPA test battery, in comparison to placebo-treated littermates. While the placebo-treated mice showed a significant progression of their phenotype within the four weeks treatment period, RD2RD2 treatment was able to slow progression of the disease phenotype and especially the motor deficits to an extent that during the four weeks treatment duration, no significant progression was observed in any of the motor experiments. Significant changes between the groups were already apparent after two treatment weeks. Further investigations on motor deficits were conducted by the modified pole test, a test measuring complex motor behaviour. Impairment of the motor skills of SOD1*G93A mice progressed slowly but significantly in the placebo group, but not in the RD2RD2-treated group. A limitation of the study is certainly the limited treatment duration, which is attributed to the use of the Alzet minipumps, not allowing statements beyond the four weeks treatment. Additionally, the use of only female mice could possibly have an impact on the variability of the results, as it is described for SOD1*G93A mice on a C57BL/6 background, for the rotarod performance [41].

In addition to the clinical symptoms like motor deficits, neuroinflammation is one prominent pathological hallmark of ALS, and previous studies demonstrated the key role of activated astrocytes and microglia cells in disease progression [17,21]. RD2RD2 treatment led to decreased levels of gliosis in the brain stem and reduced levels of astrogliosis in the lumbar spinal cord in RD2RD2-treated SOD1*G93A mice vs. placebo-treated littermates, indicating that RD2RD2 treatment efficiently reduced neuroinflammation. Furthermore, we investigated the density of neurons in the motor cortex and brain stem. Motor cortex neurons regulate the control of motor output and selectively degenerate in ALS [47]. During disease progression, degeneration of neurons of the motor cortex and brain stem causes muscle weakness and deficits in motor performance. Analysis of the neuronal nuclei in brain stem and motor cortex revealed a loss of neurons in SOD1*G93A mice in comparison to non-transgenic littermates, as described previously by others [48,49]. However, there was a significantly higher density of neurons in both the motor cortex and brain stem of RD2RD2- vs. placebo-treated mice. This supports our assumption that RD2RD2 has a beneficial effect on neuronal survival in this mouse model of ALS.

The pharmacokinetic characteristics including blood–brain barrier (BBB) penetration ability for RD2RD2 have not been determined, yet. However, for closely related compounds (RD2 and other derivatives), the pharmacokinetic profile and sufficient uptake into the brain have already been demonstrated [37,50,51,52]. Additionally, the BBB is known to be compromised in SOD1*G93A mice [53,54] allowing higher brain penetration as compared to wildtype mice. Therefore, we assume sufficient brain uptake for RD2RD2 but cannot exclude a major effect of the substance on the peripheral immune system, which in turn may attenuate inflammation systemically.

Because the target of RD2RD2 is not known yet, we speculate hypothetically about potential targets. RD2RD2 was developed for the direct disassembly of Aβ peptide oligomers into monomers. It was shown that in a double transgenic mouse line that overexpresses human SOD1*G93A and human APP-C100, the overexpression of Aβ is associated with an acceleration of onset of motor impairment and SOD1*G93A aggregation [55]. Thus, it could possibly be that RD2RD2 acts via its original mode of action. However, the link between SOD1 and Aβ was only shown in mice that overexpress both human proteins. In our study the mice overexpress only a mutant version of human SOD1, but have endogenous murine Aβ, thus it is very unlikely that murine Aβ is the main target of RD2RD2 in this experimental set up. RD2RD2 contains 10 arginine residues, which is why it can be assigned to the class of cationic arginine-rich cell-penetrating peptides (CPP). It has been reported that CPPs display anti-inflammatory and neuroprotective properties in traumatic brain injury and stroke [56,57]. In this context it has been hypothesised that the neuroprotective effect of CPPs is mediated by internalisation of neuronal cell surface structures like transporters or ion channels, as a result of endocytosis thereby leading for example to a reduction of calcium ion influx associated with excitotoxicity and other receptor-mediated neurotoxicity inducing signalling pathways [58]. After internalisation CPPs possibly also act on other targets like proprotein convertase (PCs), as it was shown that some poly-arginine peptides were potent inhibitors of PCs of the constitutive secretory pathway (PC5/6, PC7and furin) [59]. The inhibition of furin could possibly protect against neuronal cell death induced by activated *N*-methyl-d-aspartate (NMDA) receptors, as it was shown for two furin inhibitor by *Yamada* et al. [60]. Other hypothetical targets could possibly be receptors like the ionotropic P2X and metabotropic P2Y purinergic receptor, which can activate microglia after induction via ATP which is released by dying and abnormally functioning neurons and which play a key role in neuroinflammatory processes [61]. For example, the P2X_7_ receptor isoform was found to be localised on glia cells [62,63] and activation of the P2X_7_ receptor leads to an inflammatory stimulus of pro-inflammatory cytokines like interleukin-1β, a key mediator in neurodegeneration [64,65]. Whereas the loss of function of the P2X_7_ receptor in C57BL/6-J mice resulted in a substantially attenuated inflammatory response [66]. Moreover, the inhibition of the receptor with an antagonist led to an improved phenotype progression in SOD1*G93A mice [67]. Non-specific binding of RD2RD2 to the purinergic P2X receptor might also be a possible explanation for our results.

In summary, RD2RD2 demonstrated therapeutic efficacy in the SOD1*G93A ALS mouse model. Treatment with RD2RD2 led to a reduction of activated glia cell levels in the brain stem and lumbar spinal cord in SOD1*G93A mice. Analysis of neurons in the brain revealed a neuroprotective function of RD2RD2 in treated mice. The phenotype progression of SOD1*G93A mice was halted at treatment start, as there was no significant progression during the treatment period. So far, the direct target of RD2RD2 is unknown. Therefore, future studies will focus on how RD2RD2 affects neuroinflammation and ALS pathogenesis. However, based on the presented results, we conclude that RD2RD2 is a potential therapeutic candidate against ALS.

## 4. Materials and Methods

### 4.1. Ethical Approval

All applicable international and national guidelines for the care and use of animals were followed. All procedures performed in this study were in accordance with the ethical standards of the institution or practice at which the study was conducted. All animal experiments, including details on design, protocols and analysis plan, were performed in accordance with the Animal Research: Reporting of In Vivo Experiments (ARRIVE) guidelines, the German Law on the protection of animals (TierSchG §§ 7-9) and with permit from the local ethics committee (Landesamt für Natur, Umwelt und Verbraucherschutz Nordrhein-Westfalen (LANUV), North Rhine-Westphalia, Germany; AZ 84-02.04.2015.A106 and AZ 84-02.04.2014.A423). Experimental protocols, including study design and analysis plan, were reviewed and approved by the animal welfare commission of the local authority (LANUV, North Rhine-Westphalia, Germany).

### 4.2. Animals

Transgenic SOD1*G93A mice and their non-transgenic littermates were bred from male mice transgenic for human SOD1*G93A (B6.Cg-Tg(SOD1*G93A)1Gur/J mice, carrying a high copy number of the transgene, purchased from JAX (The Jackson Laboratory, ME, USA)) and female C57BL/6-J mice obtained from CRIVER (Charles River Laboratories, Sulzfeld, Germany). For breeding only male transgenic SOD1*G93A mice were used, as transgenic SOD1*G93A females are poor breeders. Due to this limitation only sufficiently large groups of female mice were available for this treatment study. Progenies were analysed for presence of the human SOD1 gene by quantitative PCR, as previously described [46]. Copy numbers of the transgene were checked by calculation of the delta cycle threshold (∆CT) = CT_internal control_ − CT_gene of interest_. Female SOD1*G93A mice with a high copy number of the transgene were selected for stratified randomisation into equally groups. Housing of the animals was under the same terms at the animal facility of the Forschungszentrum Jülich as described previously [35,68].

### 4.3. Study Drug

The d-peptide RD2RD2 (amino acid sequence: ptlhthnrrrrrptlhthnrrrrr, 3.2 kDa) was purchased from peptides & elephants (Potsdam, Germany) and Cambridge Peptides (Cambridge Peptides, Birmingham, UK) as lyophilized powder with a minimal purity of 95%. The peptide consists of 24 d-enantiomeric amino acid residues with its C-terminus being amidated.

### 4.4. Treatment

Twelve weeks old female SOD1*G93A mice and their non-transgenic littermates were treated intraperitoneally by use of Alzet osmotic minipumps (Alzet osmotic minipumps, model #1004, Alzet, USA). SOD1*G93A mice were treated with 10 mg per minipump equalling 19 mg/kg/d RD2RD2 (*n* = 12) or with physiological saline at pH 7.0 (placebo *n* = 10) as control group. Non-transgenic littermates (ntg = 13) were treated intraperitoneally with placebo exactly like the transgenic placebo group. The RD2RD2 dosage was chosen based on successful former experiments with related compounds. RD2RD2 was dissolved in sterile physiological saline at pH 7.0 and placed in minipumps for 24 h prior to implantation. The next day, the pumps were implanted intraperitoneally. In short, mice were anaesthetised with isoflurane, the skin and the muscle layer below was cut in the midline and the pump was inserted in the abdominal cavity. Following placement of the pump, the wound was sutured. All mice received three days of carprofen treatment after surgery (day of surgery plus two days after). Mice were monitored regularly for possible complications related to the surgical intervention and were medically attended. The sutures were removed aseptically approximately 7 to 10 days after surgery.

### 4.5. Body Weight of SOD1*G93A Mice

The weight of the SOD1*G93A animals was recorded at least three times per week beginning prior to pump implantation. Weighing was always performed between 8 a.m. and 9 a.m. to avoid diurnal variations.

### 4.6. Behavioural Assessment

SOD1*G93A mice were tested longitudinally in different behavioural set ups (SHIRPA and modified pole test). Each behavioural test of the SOD1*G93A mice was performed before treatment (baseline measurements) and one week after the implantation (first trial day: 8 d ± 1 d after implantation; criteria: general health, i.e., weight gain, look of fur, posture, and motor activity). The experimenter was blind to genotype or treatment. All tests were carried out at the same time of the day. Before each test, all mice were allowed to habituate in a single cage for 30 min. All mice were observed daily for disease progression.

#### 4.6.1. Phenotype Assessment

The primary screen of the SHIRPA test battery was used to assess the phenotype [39,40]. This test consisted of the following subtests, which are scored by the experimenter and summed up to an individual SHIRPA score: restlessness, alertness, startle response, pinna reflex, corneal reflex, touch response, pain response, grooming, and apathy, abnormal body carriage, abnormal gait, loss of righting reflex, forelimb placing reflex, hanging behaviour, hind limb tremor. The last seven tests mentioned above represent motor abilities of the mice and are additionally summed up to a motor score. Mice were individually tested and scored in an arena of 42.5 cm × 18.0 cm × 26.5 cm (L × H × W). Scoring was defined from 0 (similar to ntg littermates) to 3 (extremely abnormal from ntg littermates).

#### 4.6.2. Modified Pole Test

The modified pole test [68] is a sensitive functional test to measure early changes in the motor behaviour of the SOD1*G93A mice. The following modifications were realised: The mice were placed with the head downwards instead of upwards on a vertical pole (height 50 cm, diameter 1.2 cm, rough-surfaced) and their movement downwards was rated. The runs were scored from 0 to 3 (0 continuous run, 1 part-way runs, 2 slipping downwards and 3 falling down). This procedure was performed three times and the sum of all three scores was used for analysis.

#### 4.6.3. Disease Onset

To monitor the disease progression of the SOD1*G93A mice, all animals were inspected daily for signs of motor deficits. Disease onset was determined after *Mead* et al. in 2011 [41] if the following criteria were both met: *“Point at which defects in hind limb splay and enhanced tremor were observed with a score of at least 1 in each category”*.

### 4.7. Tissue Collection

At the end of the study, SOD1*G93A and non-transgenic mice were sacrificed for histopathological analysis. Brains and spinal cords of all mice were removed and snap frozen in −80 °C isopentane. Sagittal brain sections of 20 µm were cut using a cryotome (Leica Biosystems Nussloch GmbH, Wetzlar, Germany). In addition, 12 µm transversal sections of the lumbar spinal cord were harvested. The left brain hemisphere and the lumbar spinal cord (L1-L5 tract) were used for immunohistological analysis. The lumbar region of the spinal cord was identified as described previously [69,70]. Furthermore, the muscle *M. gastrocnemius* of both hind limbs were harvested along with brain and spinal cord tissues and weighted using a micro scale. Afterward, the muscles were incubated in 4% paraformaldehyde for 1 h, followed by an incubation in 30% sucrose solution for 2 h. Muscles were snap frozen in −80 °C isopentane and then cut in transversal sections using a cryotome (Leica Biosystems Nussloch GmbH, Wetzlar, Germany). Samples were stored at −80 °C until further processing.

### 4.8. Immunohistochemical Staining

Gliosis (antibodies GFAP for astrocytes and CD11b for microglia) and neuronal survival (antibody NeuN for mature neurons) were assessed by immunohistochemical analysis. Tissue sections were fixed with 4% paraformaldehyde and treated with 70% formic acid for antigen retrieval. The sections were rinsed and treated with 3% H_2_O_2_ in methanol for elimination of endogenous peroxidases. After a further washing step, sections were incubated with the primary antibody overnight at 4 °C in a humid chamber (GFAP: DAKO Agilent Technologies, Santa Clara, USA; NeuN: Merck Millipore, Darmstadt, Germany) or for 2 h at room temperature (RT) (CD11b: Abcam, Cambridge, UK). Primary antibodies were diluted 1:1000 in tris buffered saline with 1% Triton X-100 (TBST) with 1% bovine serum albumin (BSA) (GFAP and NeuN) or 1:2000 in tris buffered saline (TBS) with 1% BSA (CD11b).

Afterwards, immunolabelled sections with GFAP, NeuN and CD11b antibody were rinsed and incubated with biotinylated secondary anti-mouse or anti-rabbit antibody (1:1000 in TBST with 1% BSA (GFAP and NeuN) or in TBS with 1% BSA (CD11b), Sigma Aldrich, Germany) for 2 h at RT followed by 3, 3’-Diaminobenzidine enhanced with saturated nickel ammonium sulfate solution.

Muscle degeneration of the *M. gastrocnemius* were visualised by haematoxylin and eosin (H&E) staining. Thawed tissue sections were fixed with 4% paraformaldehyde for 10 min and rinsed with tap water. The sections were placed in a staining cuvette and treated with haematoxylin for three minutes at RT. After another tap water rinse, sections were incubated in eosin solution for two minutes at RT, followed by a further rinse in tap water. Immunohistochemical sections of brain, lumbar spinal cord and *M. gastrocnemius* were mounted with DPX Mountant medium (Sigma Aldrich, Germany) after washing in an ascending alcohol series.

### 4.9. Quantification

Images of SOD1*G93A sections were taken with a LMD6000 microscope (Leica Camera, Wetzlar, Germany) and LAS 4.0 software (Leica, Wetzlar, Germany). Immunoreactive microglial cells (antibody CD11b) and astrogliosis (antibody GFAP) were determined as percentage area (%) of the neuropil occupied by GFAP or CD11b immunoreactivity or of neuron nuclei (antibody NeuN) as count per stained area using ImageJ (National Institute of Health, Bethesda, USA) and CellProfiler Analyst (Broad Institute, Boston, USA) [71,72]. To avoid deviations in the analysis of the region of interest in the brain stem and motor cortex, a standard circle or rectangle was created with the ImageJ program. The whole tissue sections of the lumbar spinal cord were freehand selected with the ImageJ program and quantified. Histopathology analyses in SOD1*G93A were carried out in brain stem and lumbar spinal cord. The CD11b immunoreactive area was analysed in the brain stem and lumbar spinal cord (brain stem: 3 to 4 slides per mouse, ntg *n* = 13, RD2RD2 *n* = 12, placebo *n* = 10 and lumbar spinal cord: 4 to 8 slides per mouse, ntg *n* = 11, RD2RD2 *n* = 11, placebo *n* = 10). The GFAP immunoreactive area was analysed in the brain stem and lumbar spinal cord (brain stem: 4 to 6 slides per mouse, ntg *n* = 13, RD2RD2 *n* = 11, placebo *n* = 9 and lumbar spinal cord: 4 to 8 slides per mouse, ntg *n* = 12, RD2RD2 *n* = 10, placebo *n* = 8). NeuN counts were analysed in the brain stem and motor cortex layers 2/3 and 5 (brain stem: 3 to 5 slides per mouse, ntg *n* = 11, RD2RD2 *n* = 11, placebo *n* = 8 and motor cortex: 4 to 5 slides per mouse, ntg *n* = 10, RD2RD2 *n* = 10, placebo *n* = 10).

### 4.10. Statistics

Statistical analyses were performed using SigmaPlot Version 11 (Systat Software, Germany) and GraphPad Prism 8 (GraphPad Software Inc., USA) was used for the graphic illustrations. Presentation of data as mean ± SEM (behavioural tests and histochemical analysis), *p* > 0.05 was considered as not significant (ns). Normal distribution of data was tested by use of Shapiro–Wilk normality test (SigmaPlot Version 11, Systat Software, Germany). Two-way repeated measurement (RM) ANOVA with Fisher’s Least Significant Difference (LSD) post hoc analysis was used to analyse the results of the behavioural tests of SOD1*G93A mice (body weight, SHIRPA test, modified pole test). One-way measurement ANOVA with Fisher’s least significant difference (LSD) post hoc analysis was used to analyse the results of the histochemical analysis.

## Figures and Tables

**Table 1:** Evaluation of immunolabelling in brain and lumbar spinal cord of SOD1*G93A mice revealed a decrease of gliosis after treatment with RD2RD2. Data is presented as mean ± SEM. Statistical calculations were conducted by one-way ANOVA with Fisher’s LSD post hoc analysis. Lozenges (^#^) and asterisks (*) indicate a significance between treatment groups (ntg vs. RD2RD2 or ntg vs. placebo: ^#^ *p* = 0.05, ^##^ *p* = 0.01, ^###^ *p* < 0.001 and RD2RD2 vs. placebo: * *p* = 0.05, *** *p* < 0.001).

IR ^1^	Area	ntg	RD2RD2	Placebo	Statistic (One-Way ANOVA Analysis of Variance)
CD11b (%)	brain stem	1.21 ± 0.13	2.41 ± 0.28 ^#,^ ***	4.29 ± 0.54 ^###^	F(2,32) = 22.02, *p* < 0.001 ntg vs. RD2RD2 *p* = 0.011 ntg vs. placebo *p* < 0.001 RD2RD2 vs. placebo *p* < 0.001
	lumbar spinal cord	0.46 ± 0.07	1.00 ± 0.23	1.57 ± 0.44 ^##^	F(2,29) = 3.96, *p* = 0.030 ntg vs. RD2RD2 *p* = 0.175 (ns) ntg vs. placebo *p* = 0.009 RD2RD2 vs placebo *p* = 0.156 (ns)
GFAP (%)	brain stem	1.43 ± 0.16	3.23 ± 0.37 ^##,^ *	4.68 ± 0.88 ^###^	F(2,30) = 11.62, *p* < 0.001 ntg vs. RD2RD2 *p* = 0.009 ntg vs. placebo *p* < 0.001 RD2RD2 vs. placebo *p* = 0.049
	lumbar spinal cord	0.73 ± 0.08	1.69 ± 0.4 *	3.16 ± 0.6 ^###^	F(2,27) = 10.93, *p* < 0.001 ntg vs. RD2RD2 *p* = 0.060 (ns) ntg vs. placebo *p* < 0.001 RD2RD2 vs. placebo *p* = 0.011
NeuN (counts)	brain stem	450 ± 42.3	406 ± 31.8 ^##,^ *	343 ± 46.4 ^###^	F(2,27) = 1.66, *p* = 0.208 ntg vs. RD2RD2 *p* = 0.002 ntg vs. placebo *p* < 0.001 RD2RD2 vs. placebo *p* = 0.021
	motor cortex	947 ± 42.8	924 ± 32.9 *	784 ± 42.6 ^##^	F(2,27) = 4.92, *p* = 0.015 ntg vs. RD2RD2 *p* = 0.677 (ns) ntg vs. placebo *p* = 0.007 RD2RD2 vs. placebo *p* = 0.020

^1^ IR: immunoreactivity.

## Data Availability

The datasets used and/or analysed during the current study are available from the corresponding author on reasonable request.

## Supplementary Materials

The following are available online, Figure S1: Analysis of neuroinflammation in cortex and hippocampus of RD2RD2-treated APP/PS1 mice; Figure S2: Treatment with RD2RD2 significantly reduced levels of inflammatory markers in the plasma of APP/PS1 mice; Figure S3: Phenotype assessment of SOD1*G93A mice and their non-transgenic littermates; Table S1. Treatment with RD2RD2 significantly reduced gliosis in APP/PS1 mice.; Table S2: Cytokine assay of RD2RD2- and placebo-treated APP/PS1 mice. References from Supplementary materials [71,73,74,75].

## Sample Availability

Samples of the compound are available from the corresponding author on reasonable request.

## References

[1] Cacquevel M., Lebeurrier N., Chéenne S., Vivien D. (2004). Cytokines in neuroinflammation and Alzheimer’s disease. Curr. Drug Targets.

[2] Maeda J., Ji B., Irie T., Tomiyama T., Maruyama M., Okauchi T., Staufenbiel M., Iwata N., Ono M., Saido T.C. (2007). Longitudinal, Quantitative Assessment of Amyloid, Neuroinflammation, and Anti-Amyloid Treatment in a Living Mouse Model of Alzheimer’s Disease Enabled by Positron Emission Tomography. J. Neurosci..

[3] Pgostinho P., Rodrigo A.C., Catarina O. (2010). Neuroinflammation, oxidative stress and the pathogenesis of Alzheimer’s disease. Curr. Pharm. Des..

[4] Heneka M.T., Carson M.J., El Khoury J., E Landreth G., Brosseron F., Feinstein D.L., Jacobs A.H., Wyss-Coray T., Vitorica J., Ransohoff R.M. (2015). Neuroinflammation in Alzheimer’s disease. Lancet Neurol..

[5] Mitchell J., Borasio G. (2007). Amyotrophic lateral sclerosis. Lancet.

[6] Cleveland D.W., Rothstein J.D. (2001). From charcot to lou gehrig: Deciphering selective motor neuron death in als. Nat. Rev. Neurosci..

[7] Deng H.X., Hentati A., A Tainer J., Iqbal Z., Cayabyab A., Hung W.Y., Getzoff E.D., Hu P., Herzfeldt B., Roos R.P. (1993). Amyotrophic lateral sclerosis and structural defects in Cu, Zn superoxide dismutase. Science.

[8] Rosen D.R., Siddique T., Patterson D., Figlewicz D.A., Sapp P.C., Hentati A., Donaldson D.H., Goto J., O’Regan J.P., Deng H.-X. (1993). Mutations in Cu/Zn superoxide dismutase gene are associated with familial amyotrophic lateral sclerosis. Nature.

[9] Gurney M.E., Pu H., Chiu A.Y., Dal Canto M.C., Polchow C.Y., Alexander D.D., Caliendo J., Hentati A., Kwon Y.W., Deng H.X. (1994). Motor neuron degeneration in mice that express a human Cu, Zn superoxide dismutase mutation. Science.

[10] Gurney M.E. (1997). The use of transgenic mouse models of amyotrophic lateral sclerosis in preclinical drug studies. J. Neurol. Sci..

[11] Wang L., Sharma K., Deng H.-X., Siddique T., Grisotti G., Liu E., Roos R.P. (2008). Restricted expression of mutant SOD1 in spinal motor neurons and interneurons induces motor neuron pathology. Neurobiol. Dis..

[12] Özdinler P.H., Benn S., Yamamoto T.H., Güzel M., Brown R.H., Macklis J.D. (2011). Corticospinal Motor Neurons and Related Subcerebral Projection Neurons Undergo Early and Specific Neurodegeneration in hSOD1G93A Transgenic ALS Mice. J. Neurosci..

[13] Rothstein J.D. (2009). Current hypotheses for the underlying biology of amyotrophic lateral sclerosis. Ann. Neurol..

[14] Philips T., Robberecht W. (2011). Neuroinflammation in amyotrophic lateral sclerosis: Role of glial activation in motor neuron disease. Lancet Neurol..

[15] Robberecht W., Philips T. (2013). The changing scene of amyotrophic lateral sclerosis. Nat. Rev. Neurosci..

[16] Jara J.H., Genç B., Stanford M.J., Pytel P., Roos R.P., Weintraub S., Mesulam M.M., Bigio E.H., Miller R.J., Özdinler P.H. (2017). Evidence for an early innate immune response in the motor cortex of ALS. J. Neuroinflamm..

[17] Boillée S., Yamanaka K., Lobsiger C.S., Copeland N.G., Jenkins N.A., Kassiotis G., Kollias G., Cleveland D.W. (2006). Onset and Progression in Inherited ALS Determined by Motor Neurons and Microglia. Science.

[18] Hensley K., Abdel-Moaty H., Hunter J., Mhatre M., Mou S., Nguyen K., Potapova T., Pye Q.N., Qi M., Rice H. (2006). Primary glia expressing the G93A-SOD1 mutation present a neuroinflammatory phenotype and provide a cellular system for studies of glial inflammation. J. Neuroinflamm..

[19] Patel N.S., Paris D., Mathura V., Quadros A.N., Crawford F.C., Mullan M.J. (2005). Inflammatory cytokine levels correlate with amyloid load in transgenic mouse models of Alzheimer’s disease. J. Neuroinflamm..

[20] Heneka M.T., Kummer M.P., Stutz A., Delekate A., Schwartz S., Vieira-Saecker A., Griep A., Axt D., Remus A., Tzeng T.-C. (2013). NLRP3 is activated in Alzheimer’s disease and contributes to pathology in APP/PS1 mice. Nat. Cell Biol..

[21] Lewis C.-A., Manning J., Rossi F., Krieger C. (2012). The Neuroinflammatory Response in ALS: The Roles of Microglia and T Cells. Neurol. Res. Int..

[22] Greenhalgh C.J., Hilton D.J. (2001). Negative regulation of cytokine signaling. J. Leukoc. Biol..

[23] Turner M.D., Nedjai B., Hurst T., Pennington D.J. (2014). Cytokines and chemokines: At the crossroads of cell signalling and inflammatory disease. Biochim. Biophys. Acta.

[24] Elliott J.L. (2001). Cytokine upregulation in a murine model of familial amyotrophic lateral sclerosis. Mol. Brain Res..

[25] McGeer P.L., McGeer E.G. (2002). Inflammatory processes in amyotrophic lateral sclerosis. Muscle Nerve.

[26] Jeyachandran A., Mertens B., McKissick E.A., Mitchell C.S. (2015). Type I Vs. Type II cytokine levels as a function of SOD1 G93A mouse amyotrophic lateral sclerosis disease progression. Front. Cell. Neurosci..

[27] Brooks B.R., Miller R.G., Swash M., Munsat T.L. (2000). El Escorial revisited: Revised criteria for the diagnosis of amyotrophic lateral sclerosis. Amyotroph. Lateral Scler..

[28] Miller R.G., Mitchell J.D., Moore D.H. (2012). Riluzole for amyotrophic lateral sclerosis (ALS)/motor neuron disease (MND). Cochrane Database Syst. Rev..

[29] Dorst J., Ludolph A.C., Huebers A. (2017). Disease-modifying and symptomatic treatment of amyotrophic lateral sclerosis. Ther. Adv. Neurol. Disord..

[30] Zhang T., Loschwitz J., Strodel B., Nagel-Steger L., Willbold D. (2019). Interference with Amyloid-β Nucleation by Transient Ligand Interaction. Molecules.

[31] Willbold D., Kutzsche J. (2019). Do We Need Anti-Prion Compounds to Treat Alzheimer’s Disease?. Molecules.

[32] Kutzsche J., Jürgens D., Willuweit A., Adermann K., Fuchs C., Simons S., Windisch M., Hümpel M., Rossberg W., Wolzt M. (2020). Safety and pharmacokinetics of the orally available antiprionic compound PRI-002: A single and multiple ascending dose phase I study. Alzheimer’s Dement. Transl. Res. Clin. Interv..

[33] Van Groen T., Schemmert S., Brener O., Gremer L., Ziehm T., Tusche M., Nagel-Steger L., Kadish I., Schartmann E., Elfgen A. (2017). The Aβ oligomer eliminating d-enantiomeric peptide RD2 improves cognition without changing plaque pathology. Sci. Rep..

[34] Kutzsche J., Schemmert S., Tusche M., Neddens J., Rabl R., Jürgens D., Brener O., Willuweit A., Hutter-Paier B., Willbold D. (2017). Large-Scale Oral Treatment Study with the Four Most Promising D3-Derivatives for the Treatment of Alzheimer’s Disease. Molecules.

[35] Schemmert S., Schartmann E., Zafiu C., Kass B., Hartwig S., Lehr S., Bannach O., Langen K.-J., Shah N.J., Kutzsche J. (2019). Aβ Oligomer Elimination Restores Cognition in Transgenic Alzheimer’s Mice with Full-blown Pathology. Mol. Neurobiol..

[36] Schemmert S., Schartmann E., Honold D., Zafiu C., Ziehm T., Langen K.-J., Shah N.J., Kutzsche J., Willuweit A., Willbold D. (2019). Deceleration of the neurodegenerative phenotype in pyroglutamate-Aβ accumulating transgenic mice by oral treatment with the Aβ oligomer eliminating compound RD2. Neurobiol. Dis..

[37] Leithold L.H., Jiang N., Post J., Niemietz N., Schartmann E., Ziehm T., Kutzsche J., Shah N.J., Breitkreutz J., Langen K.-J. (2016). Pharmacokinetic properties of tandem d-peptides designed for treatment of Alzheimer’s disease. Eur. J. Pharm. Sci..

[38] Elfgen A., Hupert M., Bochinsky K., Tusche M., Martin E.G.D.S.R., Gering I., Sacchi S., Pollegioni L., Huesgen P.F., Hartmann R. (2019). Metabolic resistance of the d-peptide RD2 developed for direct elimination of amyloid-β oligomers. Sci. Rep..

[39] Rogers D.C., Fisher E.M.C., Brown S.D.M., Peters J., Hunter A.J., Martin J.E. (1997). Behavioral and functional analysis of mouse phenotype: SHIRPA, a proposed protocol for comprehensive phenotype assessment. Mamm. Genome.

[40] Rogers D.C., Peters J., Martin J.E., Ball S., Nicholson S.J., Witherden A.S., Hafezparast M., Latcham J., Robinson T.L., Quilter C.A. (2001). SHIRPA, a protocol for behavioral assessment: Validation for longitudinal study of neurological dysfunction in mice. Neurosci. Lett..

[41] Mead R.J., Bennett E.J., Kennerley A.J., Sharp P., Sunyach C., Kasher P., Berwick J., Pettmann B., Battaglia G., Azzouz M. (2011). Optimised and Rapid Pre-clinical Screening in the SOD1G93A Transgenic Mouse Model of Amyotrophic Lateral Sclerosis (ALS). PLoS ONE.

[42] Cummings J., Lee G., Ritter A., Zhong K. (2018). Alzheimer’s disease drug development pipeline: 2018. Alzheimer’s Dement. Transl.Res. Clin. Interv..

[43] Miao J., Xu F., Davis J., Otte-Höller I., Verbeek M.M., Van Nostrand W.E. (2005). Cerebral Microvascular Amyloid β Protein Deposition Induces Vascular Degeneration and Neuroinflammation in Transgenic Mice Expressing Human Vasculotropic Mutant Amyloid β Precursor Protein. Am. J. Pathol..

[44] Craft J.M., Watterson D.M., Van Eldik L.J. (2006). Human amyloid β-induced neuroinflammation is an early event in neurodegeneration. Glia.

[45] Lewis E.K., Rasmussen A.L., Bennett W., King A., West A.K., Chung R.S., Chuah M.I. (2014). Microglia and motor neurons during disease progression in the SOD1G93A mouse model of amyotrophic lateral sclerosis: Changes in arginase1 and inducible nitric oxide synthase. J. Neuroinflamm..

[46] Ohgomori T., Yamasaki R., Takeuchi H., Kadomatsu K., Jinno S., Kira J.-I. (2017). Differential activation of neuronal and glial STAT3 in the spinal cord of theSOD1G93Amouse model of amyotrophic lateral sclerosis. Eur. J. Neurosci..

[47] Fogarty M.J., Noakes P.G., Bellingham M.C. (2015). Motor Cortex Layer V Pyramidal Neurons Exhibit Dendritic Regression, Spine Loss, and Increased Synaptic Excitation in the Presymptomatic hSOD1G93A Mouse Model of Amyotrophic Lateral Sclerosis. J. Neurosci..

[48] An T., Shi P., Duan W., Zhang S., Yuan P., Li Z., Wu D., Xu Z., Li C., Guo Y. (2014). Oxidative Stress and Autophagic Alteration in Brainstem of SOD1-G93A Mouse Model of ALS. Mol. Neurobiol..

[49] Solomonov Y., Hadad N., Levy R. (2016). Reduction of cytosolic phospholipase A2α upregulation delays the onset of symptoms in SOD1G93A mouse model of amyotrophic lateral sclerosis. J. Neuroinflamm..

[50] Jiang N., Leithold L.H.E., Post J., Ziehm T., Mauler J., Gremer L., Cremer M., Schartmann E., Shah N.J., Kutzsche J. (2015). Preclinical Pharmacokinetic Studies of the Tritium Labelled D-Enantiomeric Peptide D3 Developed for the Treatment of Alzheimer´s Disease. PLOS ONE.

[51] Leithold L.H.E., Jiang N., Post J., Ziehm T., Schartmann E., Kutzsche J., Shah N.J., Breitkreutz J., Langen K.-J., Willuweit A. (2015). Pharmacokinetic Properties of a Novel d-Peptide Developed to be Therapeutically Active Against Toxic β-Amyloid Oligomers. Pharm. Res..

[52] Schartmann E., Schemmert S., Ziehm T., Leithold L.H.E., Jiang N., Tusche M., Shah N.J., Langen K.-J., Kutzsche J., Willbold D. (2018). Comparison of blood-brain barrier penetration efficiencies between linear and cyclic all-d-enantiomeric peptides developed for the treatment of Alzheimer’s disease. Eur. J. Pharm. Sci..

[53] Garbuzova-Davis S., Haller E., Saporta S., Kolomey I., Nicosia S.V., Sanberg P.R. (2007). Ultrastructure of blood–brain barrier and blood–spinal cord barrier in SOD1 mice modeling ALS. Brain Res..

[54] Garbuzova-Davis S., Saporta S., Haller E., Kolomey I., Bennett S.P., Potter H., Sanberg P.R. (2007). Evidence of Compromised Blood-Spinal Cord Barrier in Early and Late Symptomatic SOD1 Mice Modeling ALS. PLOS ONE.

[55] Li Q.-X., Mok S.S., Laughton K.M., McLean C.A., Volitakis I., Cherny R.A., Cheung N.S., White A.R., Masters C.L. (2006). Overexpression of Aβ is associated with acceleration of onset of motor impairment and superoxide dismutase 1 aggregation in an amyotrophic lateral sclerosis mouse model. Aging Cell.

[56] Chiu L.S., Anderton R.S., Cross J.L., Clark V.W., Knuckey N.W., Meloni B.P. (2019). Poly-arginine Peptide R18D Reduces Neuroinflammation and Functional Deficits Following Traumatic Brain Injury in the Long-Evans Rat. Int. J. Pept. Res. Ther..

[57] Chiu L.S., Anderton R.S., Knuckey N.W., Meloni B.P. (2016). The neuroprotective potential of arginine-rich peptides for the acute treatment of traumatic brain injury. Expert Rev. Neurother..

[58] Meloni B.P., Milani D., Edwards A.B., Anderton R.S., Doig R.L.O., Fitzgerald M., Palmer T.N., Knuckey N.W. (2015). Neuroprotective peptides fused to arginine-rich cell penetrating peptides: Neuroprotective mechanism likely mediated by peptide endocytic properties. Pharmacol. Ther..

[59] Fugere M., Appel J., Houghten R.A., Lindberg I., Day R. (2007). Short polybasic peptide sequences are potent inhibitors of PC5/6 and PC7: Use of positional scanning-synthetic peptide combinatorial libraries as a tool for the optimization of inhibitory sequences. Mol. Pharmacol..

[60] Yamada M., Hayashi H., Yuuki M., Matsushima N., Yuan B., Takagi N. (2018). Furin inhibitor protects against neuronal cell death induced by activated NMDA receptors. Sci. Rep..

[61] Skaper S.D., Debetto P., Giusti P. (2009). The P2X 7 purinergic receptor: From physiology to neurological disorders. FASEB J..

[62] Yiangou Y., Facer P., Durrenberger P., Chessell I.P., Naylor A., Bountra C., Banati R.R., Anand P. (2006). COX-2, CB2 and P2X7-immunoreactivities are increased in activated microglial cells/macrophages of multiple sclerosis and amyotrophic lateral sclerosis spinal cord. BMC Neurol..

[63] D’Ambrosi N., Finocchi P., Apolloni S., Cozzolino M., Ferri A., Padovano V., Pietrini G., Carrì M.T., Volonté C. (2009). The Proinflammatory Action of Microglial P2 Receptors Is Enhanced in SOD1 Models for Amyotrophic Lateral Sclerosis. J. Immunol..

[64] Allan S.M., Rothwell N.J. (2001). Cytokines and acute neurodegeneration. Nat. Rev. Neurosci..

[65] Ferrari D., Chiozzi P., Falzoni S., Hanau S., Di Virgilio F. (1997). Purinergic Modulation of Interleukin-1β Release from Microglial Cells Stimulated with Bacterial Endotoxin. J. Exp. Med..

[66] Miller C.M., Zakrzewski A.M., Ikin R.J., Boulter N.R., Katrib M., Lees M.P., Fuller S.J., Wiley J.S., Smith N.C. (2011). Dysregulation of the inflammatory response to the parasite, Toxoplasma gondii, in P2X7 receptor-deficient mice. Int. J. Parasitol..

[67] Ruiz-Ruiz C., García-Magro N., Negredo P., Avendaño C., Bhattacharya A., Ceusters M., García A.G. (2020). Chronic administration of P2X7 receptor antagonist JNJ-47965567 delays disease onset and progression, and improves motor performance in ALS SOD1G93A female mice. Dis. Model. Mech..

[68] Dunkelmann T., Schemmert S., Honold D., Teichmann K., Butzküven E., DeMuth H.-U., Shah N.J., Langen K.-J., Kutzsche J., Willbold D. (2018). Comprehensive Characterization of the Pyroglutamate Amyloid-β Induced Motor Neurodegenerative Phenotype of TBA2.1 Mice. J. Alzheimer’s Dis..

[69] Tadros M.A., Harris B.M., Anderson W.B., Brichta A.M., Graham B.A., Callister R.J. (2012). Are all spinal segments equal: Intrinsic membrane properties of superficial dorsal horn neurons in the developing and mature mouse spinal cord. J. Physiol..

[70] Hugnot J.-P. (2013). Isolate and Culture Neural Stem Cells from the Mouse Adult Spinal Cord. Adv. Struct. Saf. Stud..

[71] Ferreira T., Rasband W. (2011). The Image J User Guide. https://imagej.net/docs/guide/146.html.

[72] McQuin C., Goodman A., Chernyshev V., Kamentsky L., Cimini B.A., Karhohs K.W., Doan M., Ding L., Rafelski S.M., Thirstrup D. (2018). CellProfiler 3.0: Next-generation image processing for biology. PLoS Biol..

[73] Holcomb L., Gordon M.N., McGowan E., Yu X., Benkovic S., Jantzen P.T., Wright K., Saad I., Mueller R., Morgan D. (1998). Accelerated Alzheimer-type phenotype in transgenic mice carrying both mutant amyloid precursor protein and presenilin 1 transgenes. Nat. Med..

[74] Webster B., Hansen L., Adame A., Crews L., Torrance M., Thal L., Masliah E. (2006). Astroglial Activation of Extracellular-Regulated Kinase in Early Stages of Alzheimer Disease. J. Neuropathol. Exp. Neurol..

[75] Hwang I.K., Choi J.H., Li H., Yoo K.-Y., Kim D.W., Lee C.H., Yi S.S., Seong J.K., Lee I.S., Yoon Y.S. (2008). Changes in Glial Fibrillary Acidic Protein Immunoreactivity in the Dentate Gyrus and Hippocampus Proper of Adult and Aged Dogs. J. Veter. Med. Sci..

