# Supplementary material for: A Novel Anti-Inflammatory d-Peptide Inhibits Disease Phenotype Progression in an ALS Mouse Model
Source: Molecules. 2021 Mar 13;26(6):1590. doi: 10.3390/molecules26061590 (PMC7999518; doi:10.3390/molecules26061590)
Supplement: Supplementary file 1 [file molecules-26-01590-s001.pdf]

Supplementary Materials

A Novel Anti-Inflammatory D-Peptide Inhibits Disease Phenotype Progression in an ALS Mouse Model

Julia Post [1], Vanessa Kogel [1], Anja Schaffrath [1], Philipp Lohmann [2], N. Jon Shah [2,3,4,5] and Karl-Josef Langen [2,6], Dieter Willbold [1,7,*], Antje Willuweit [2,*] and Janine Kutzsche [1,*]

1 Institute of Biological Information Processing, Structural Biochemistry, IBI-7, Forschungszentrum Jülich GmbH, 52425 Jülich, Germany; j.post@fz-juelich.de (J.P.); vkogel@ukaachen.de (V.K.); a.schaffrath@fz-juelich.de (A.S.)
2 Institute of Neuroscience and Medicine 4, INM-4, Medical Imaging Physics, Forschungszentrum Jülich GmbH, 52425 Jülich, Germany; p.lohmann@fz-juelich.de (P.L.); n.j.shah@fz-juelich.de (N.J.S.); k.j.langen@fz-juelich.de (K.-J.L.)
3 Institute of Neuroscience and Medicine 11, INM-11, JARA, Forschungszentrum Jülich GmbH, 52425 Jülich, Germany
4 JARA - Brain - Translational Medicine, 52074 Aachen, Germany
5 Department of Neurology, RWTH Aachen University, 52062 Aachen, Germany
6 Department of Nuclear Medicine, RWTH Aachen University, 52062 Aachen, Germany
7 Institut für Physikalische Biologie, Heinrich-Heine-Universität Düsseldorf, 40225 Düsseldorf, Germany
* Correspondence: d.willbold@fz-juelich.de (D.W.); a.willuweit@fz-juelich.de (A.W.); j.kutzsche@fz-juelich.de (J.K.); Tel.: +49-2461-619496 (J.K.)

Citation: Post, J.; Kogel, V.; Schaffrath, A.; Lohmann, P.; Shah, N.J.; Langen, K.-J.; Willbold, D.; Willuweit, A.; Kutzsche, J. A Novel Anti-Inflammatory D-Peptide Inhibits Disease Phenotype Progression in an ALS Mouse Model. *Molecules* **2021**, *26*, x. https://doi.org/10.3390/xxxxx

Academic Editor: Fernanda Borges

Received: 22 January 2021
Accepted: 11 March 2021
Published: 13 March 2021

Publisher's Note: MDPI stays neutral with regard to jurisdictional claims in published maps and institutional affiliations.

Copyright: © 2021 by the authors. Licensee MDPI, Basel, Switzerland. This article is an open access article distributed under the terms and conditions of the Creative Commons Attribution (CC BY) license (http://creativecommons.org/licenses/by/4.0/).

S1 Methods

S1.1 Ethical approval

Commissioned by the Forschungszentrum Jülich a study was performed with the contract research organisation PsychoGenics Inc. (Tarrytown, NY, USA) in accordance with PsychoGenics' Standard Operating Procedures. Procedures were approved by the Institutional Animal Care and Use Committee in accordance with the National Institute of Health Guide for the Care and Use of Laboratory Animals. Integrity of the data was ensured through a quality control process.

S1.2 Animals

The double transgenic $APP_{swe+PS1/M146L}$ (APP/PS1) AD mouse model, introduced by *Holcomb et al.* in 1998 [73], were bred at PsychoGenics Inc. (Tarrytown, NY, USA). Mice were housed in mixed-genotype and treatment groups of four female mice in a controlled environment (12/12 h light/dark cycle, humidity maintained around 50% and a room temperature between 20 °C and 23 °C). Food and water was available *ad libitum*.

S1.3 Treatment

Seven-months aged female APP/PS1 mice and their non-transgenic littermates (ntg) were treated intraperitoneally using the same procedure as described previously in the manuscript. AD mice were again treated with 10 mg per minipump equalling 14 mg/kg/d RD2RD2 (n = 15) or with physiological saline at pH 7.0 (placebo n = 15 and as control group ntg n = 13).

S1.4 Plasma and tissue collection

After four weeks of treatment, APP/PS1 and non-transgenic mice were deeply anaesthetised and monitored for loss of reflexes in which all the responses to external stimuli cease (verified by a toe pinch). The final collection of blood was done by terminal cardiac puncture. All blood samples were collected in K_2EDTA tubes and kept on ice for short-term storage. Within 15 min of blood collection, tubes were centrifuged for 10 min at 2.000 g in a refrigerated centrifuge. The supernatant (plasma) was extracted using a pipette and transferred into pre-labelled tubes. Samples were stored at - 80°C.

Following blood collections, brains of APP/PS1 and non-transgenic mice were harvested and post-immersion fixed in 4% paraformaldehyde in PBS, pH 7.4 at 4°C for three days. Brains were cut saggitally in 40 µm sections using a vibratome (Leica Biosystems Nussloch GmbH, Wetzlar, Germany). Sections were stored in cryoprotective media (PBS with 30% ethylene glycol, 30% glycerol) until further processing.

S1.5 Cytokine assay

Plasma samples from the transgenic APP/PS1 mice were measured using a Bio-Plex MAP kit (Bio-Rad Laboratories Inc., CA, USA). PsychoGenics Inc. (Tarrytown, NY, USA) carried out the measurement of the plasma samples of transgenic APP/PS1 mice (RD2RD2 n = 15 and placebo n = 15). The assay was performed according to manufacturer's protocol. The plasma samples were examined for seven specific inflammation markers: interleukin-1 beta (IL-1β), interleukin-6 (IL-6), interleukin-10 (IL-10), interleukin-12 heterodimer p70 (IL-12p70), interferon gamma (IFN-γ), tumor necrosis factor alpha (TNF-α) and the C-X-C motif ligand 1 (CXCL1). In general, values below the limit of detection (LoD) were excluded from analysis. Inflammatory marker data were presented as picograms per millilitre (pg/mL).

S1.7 Immunohistochemical staining

Gliosis (ionized calcium binding adaptor molecule 1 (Iba1) antibody for microglia and glial fibrillary acidic protein (GFAP) antibody for astrocytes) of eight months old APP/PS1 mice was assessed by immunohistochemical analysis. Immunolabelling was performed on free-floating sections. The sections were rinsed in PBS and incubated in 1% Triton X-100, 10% H_2O_2 in PBS for 20 min at RT. After another PBS rinse, sections were blocked in 10% normal horse serum in PBS for 1 h at RT. Primary antibodies were solved in PBS (anti-Iba1, 1:500, Abcam, Cambridge, UK; anti-GFAP, 1:2000, DAKO Agilent Technologies, Santa Clara, USA) and brain sections were incubated overnight at 4°C in a humid chamber.

Afterward, immunolabelled sections with GFAP and Iba1 antibody were rinsed and incubated with biotinylated secondary anti-mouse antibody (1:1000 in PBST with 1% BSA (Sigma Aldrich, Germany)) for 2 h at RT followed by 3, 3′-Diaminobenzidine. Immunohistochemical sections were mounted with DPX Mountant medium (Sigma Aldrich, Germany) after washing in an ascending alcohol series.

S1.8 Quantification

Immunolabelled sections with GFAP and Iba1 of APP/PS1 mice were analysed with a digital Olympus BX50 microscope (Olympus America Inc., Center Valley, USA). Histopathology analyses in APP/PS1 were carried out in the hippocampus and cortex region of the brain (ntg n = 4, RD2RD2 n = 8 and placebo n = 8). A total of three sections (4 images per section) were analysed with ImageJ (NIH) to estimate the immunoreactive area of microglial cells per unit (mm^2) by Iba1 and GFAP immunoreactivity (astrogliosis) as optical density (OD) [71, 74, 75].

S1.9 Phenotype assessment

In our pilot study, four weeks aged SOD1*G93A (B6.Cg-Tg(SOD1*G93A)1Gur/J) mice and their non-transgenic littermates were tested in different behavioural set ups. For phenotype assessment, the SHIRPA test battery and the modified pole test were performed as described in the method section of the manuscript.

S1.10 Statistics

Statistical analysis were performed using SigmaPlot Version 11 (Systat Software, Germany). GraphPad Prism 8 (GraphPad Software Inc., USA) was used for the graphic illustrations. Presentation of data as mean ± SEM (behavioural tests, histochemical and biochemical analysis), $p > 0.05$ was considered as not significant (ns). Normal distribution of data was tested by use of Shapiro-Wilk normality test (SigmaPlot Version 11, Systat Software, Germany). One-way measurement ANOVA with LSD post hoc analysis was used to analyse the results of the histochemical analysis (quantification of APP/PS1 samples), biochemical analysis (cytokine assay of APP/PS1 samples) and behavioural tests of SOD1*G93A mice (SHIRPA test and modified pole test).

S2 Figures

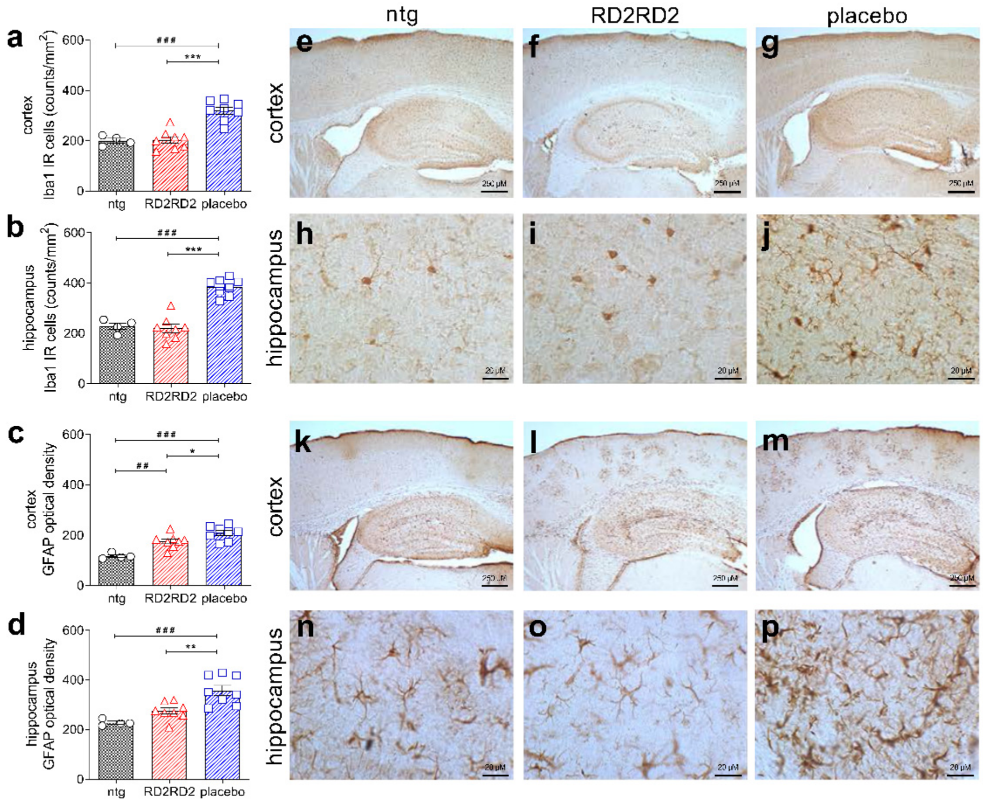

Figure S1. Analysis of neuroinflammation in cortex and hippocampus of RD2RD2-treated APP/PS1 mice. Treatment with RD2RD2 significantly reduced both the number of activated microglia (antibody Iba1) and of activated astrocytes (antibody GFAP) in the cortex and hippocampus of APP/PS1 mice. Presentation of the analysed cells and brain regions are given on the right (microglia: **a**, **e**-**g** = cortex and **b**, **h**-**j** = hippocampus; astrocytes: **c**, **k**-**m** = cortex and **d**, **n**-**p** = hippocampus). Data is presented as mean ± SEM. Statistical calculations were conducted by one-way ANOVA with Fisher's LSD post hoc analysis, ntg n = 4, RD2RD2 n = 8 and placebo n = 8. Lozenges (#) and asterisks (*) indicate a significance between treatment groups (ntg vs RD2RD2 or ntg vs placebo: ## $p < 0.01$, ### $p < 0.001$ and RD2RD2 vs placebo: * $p = 0.05$, ** $p = 0.01$, *** $p < 0.001$). IR: immunoreactivity. Circles: placebo-treated ntg; triangles: RD2RD2-treated SOD1*G93A mice and squares: placebo-treated SOD1*G93A mice.

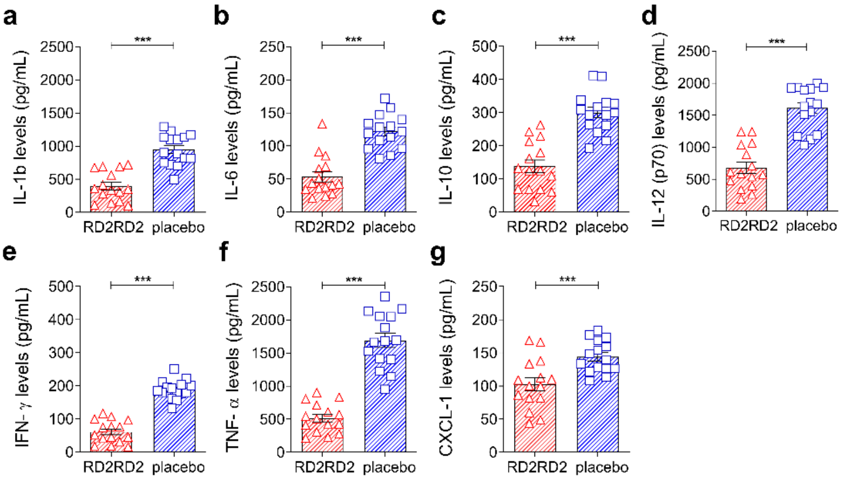

Figure S2. Treatment with RD2RD2 significantly reduced levels of inflammatory markers in the plasma of APP/PS1 mice. A Bio-Plex Map kit was used to analyse a possible change of inflammatory cytokines at the end of the study. Data revealed a significant reduction in all cytokines due to RD2RD2 treatment in comparison to placebo treatment (**a-g**). Cytokine concentrations are given in picogram per milliliter (pg/mL). Data is presented as mean ± SEM. Statistical calculations were conducted by one-way ANOVA with Fisher's LSD post hoc analysis, RD2RD2 n = 15 and placebo n = 15 for each cytokine. Asterisks (*) indicate a significance between treatment groups (RD2RD2 vs placebo: *** $p < 0.001$). Circles: placebo-treated ntg; triangles: RD2RD2-treated SOD1*G93A mice and squares: placebo-treated SOD1*G93A mice.

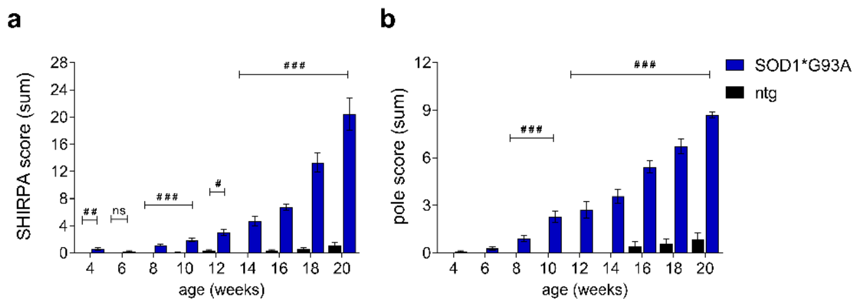

Figure S3. Phenotype assessment of SOD1*G93A mice and their non-transgenic littermates. The SHIRPA test battery (**a**) and the modified pole test (**b**) were used to evaluate the phenotypic development of SOD1*G93A mice. Four weeks aged mice were tested regularly every second week up to an age of 20 weeks. At an age of 10 weeks seven animals of each group went into analysis of hind limb muscles and the phenotype assessments went on with the remaining seven animals. Data is presented as mean ± SEM. Statistical calculations were conducted by one-way ANOVA with Fisher's LSD post hoc analysis, ntg n = 14 and SOD1*G93A n = 14 (weeks 4 to 10) and ntg n = 7 and SOD1*G93A n = 7 (weeks 12 to 20).

S3 Tables

Table S1. Treatment with RD2RD2 significantly reduced gliosis in APP/PS1 mice. Analysis of activated glia cells in AD mice indicate a significant change in the neuroinflammatory pathology after intraperitoneal treatment with RD2RD2 compared to placebo-treated mice. Data is presented as mean ± SEM. Statistical calculations were conducted by one-way ANOVA with Fisher's LSD post hoc analysis. Lozenges (#) and asterisks (*) indicate a significance between treatment groups (ntg vs RD2RD2 or ntg vs placebo: ### $p < 0.001$ and RD2RD2 vs placebo: * $p = 0.05$, ** $p = 0.01$, *** $p < 0.001$). IR: immunoreactivity, OD: optical density

IR	Area	ntg	RD2RD2	placebo	Statistic (one-way ANOVA Analysis of Variance)
Iba1 (counts)	cortex	228 ± 13.4	203 ± 13.2 ***	320 ± 14.1 ###	$F(2,17) = 25.57$, $p < 0.001$<br>ntg vs RD2RD2 $p = 0.876$ (ns)<br>ntg vs placebo $p < 0.001$<br>RD2RD2 vs placebo $p < 0.001$
	hippocampus	200 ± 9.92	220 ± 16.2 ***	383 ± 12.1 ###	$F(2,17) = 42.28$, $p < 0.001$<br>ntg vs RD2RD2 $p = 0.722$ (ns)<br>ntg vs placebo $p < 0.001$<br>RD2RD2 vs placebo $p < 0.001$
GFAP (OD)	cortex	117 ± 5.66	176 ± 9.66 ###, *	208 ± 9.92 ###	$F(2,17) = 17.04$, $p < 0.001$<br>ntg vs RD2RD2 $p = 0.002$<br>ntg vs placebo $p < 0.001$<br>RD2RD2 vs placebo $p = 0.021$
	hippocampus	228 ± 6.29	275 ± 12.4 **	357 ± 20.5 ###	$F(2,17) = 13.70$, $p < 0.001$<br>ntg vs RD2RD2 $p = 0.097$ (ns)<br>ntg vs placebo $p < 0.001$<br>RD2RD2 vs placebo $p = 0.001$

Table S2. Cytokine assay of RD2RD2- and placebo-treated APP/PS1 mice. A Bio-Plex Map kit was used to analyse a possible change of inflammatory cytokines at the end of the study. Treatment with RD2RD2 significantly reduced levels of inflammatory markers in the blood of APP/PS1 mice. Cytokine concentrations are given in picogram per milliliter (pg/mL). Data is presented as mean ± SEM. Statistical calculations were conducted by one-way ANOVA with Fisher's LSD post hoc analysis, RD2RD2 n = 15 and placebo n = 15 for each cytokine. Asterisks (*) indicate a significance between treatment groups (RD2RD2 vs placebo: *** p < 0.001).

Marker (pg/mL)	RD2RD2	placebo	Statistic (one-way ANOVA Analysis of Variance)
IL-1β	394 ± 57.6 ***	950 ± 60.4	$F(1,28) = 44.52$, $p < 0.001$<br>RD2RD2 vs placebo $p < 0.001$
IL-6	53.6 ± 7.87 ***	122 ± 6.75	$F(1,28) = 43.46$, $p < 0.001$<br>RD2RD2 vs placebo $p < 0.001$
IL-10	139 ± 18.9 ***	300 ± 16	$F(1,28) = 42.74$, $p < 0.001$<br>RD2RD2 vs placebo $p < 0.001$
IL-12p70	677 ± 88.5 ***	1612 ± 87.3	$F(1,28) = 56.55$, $p < 0.001$<br>RD2RD2 vs placebo $p < 0.001$
INF-γ	61 ± 8.87 ***	189 ± 7.34	$F(1,28) = 123.56$, $p < 0.001$<br>RD2RD2 vs placebo $p < 0.001$
CXCL-1	103 ± 9.79 ***	144 ± 6.25	$F(1,28) = 12.79$, $p = 0.001$<br>RD2RD2 vs placebo $p = 0.001$
TNF-α	517 ± 57.3 ***	1689 ± 107	$F(1,28) = 93.32$, $p < 0.001$<br>RD2RD2 vs placebo $p < 0.001$

S4 References

71. Ferreira, T. & Rasband, W. The Image J User Guide. **2011**.

73. Holcomb, L. et al. Accelerated Alzheimer-type phenotype in transgenic mice carrying both mutant amyloid precursor protein and presenilin 1 transgenes. *Nat. Med.* **1998**, 4, 97-100, 10.1038/nm0198-097.

74. Webster, B. et al. Astroglial Activation of Extracellular-Regulated Kinase in Early Stages of Alzheimer Disease. *J. Neuropathol. Exp. Neurol.* **2006**, 65, 142-151, 10.1097/01.jnen.0000199599.63204.6f.

75. Hwang, I. K. et al. Changes in glial fibrillary acidic protein immunoreactivity in the dentate gyrus and hippocampus proper of adult and aged dogs. *J Vet Med Sci* **2008**, 70, 965-969, 10.1292/jvms.70.965.
